# Association Between Hair Diseases and COVID-19 Pandemic-Related Stress: A Cross-Sectional Study Analysis

**DOI:** 10.3389/fmed.2022.876561

**Published:** 2022-05-12

**Authors:** Ashrafur Rahaman Mahadi, Md. Abdur Rafi, Tasnim Shahriar, Senjuti Seemanta, Md. Golam Rabbani, Munjarin Akter, Mahabubul Islam Majumder, M. Tasdik Hasan

**Affiliations:** ^1^Central Medical College, Cumilla, Bangladesh; ^2^Public Health Foundation, Dhaka, Bangladesh; ^3^Rajshahi Medical College, Rajshahi, Bangladesh; ^4^Khulna Medical College, Khulna, Bangladesh; ^5^M Abdur Rahim Medical College, Dinajpur, Bangladesh; ^6^International Center for Diarrhoeal Disease Research, Dhaka, Bangladesh; ^7^Delta Medical College, Dhaka, Bangladesh; ^8^Department of Public Health, State University of Bangladesh, Dhaka, Bangladesh; ^9^Department of Primary Care & Mental Health, University of Liverpool, Liverpool, United Kingdom

**Keywords:** hair fall diseases, telogen effluvium, alopecia areata, seborrheic dermatitis, COVID-19 stress

## Abstract

**Introduction:**

Psychological stress from the ongoing coronavirus disease 2019 (COVID-19) pandemic can potentially aggravate the course of several stress-sensitive skin and hair diseases. This study aimed to determine the potential association of COVID-19 stress with hair diseases, such as telogen effluvium (TE), alopecia areata (AA), and seborrheic dermatitis (SD), among medical students in Bangladesh.

**Methods:**

This online-based cross-sectional study was conducted among 404 medical students of Bangladesh using a self-administered questionnaire, including sociodemographic information, status of hair diseases (i.e., TE, AA, and SD), COVID-19 fear scale, impact of event scale specific for COVID-19 (IES-COVID-19), and COVID-19 student stress questionnaire (CSSQ) scale, to determine pandemic-related stress. The logistic regression model was used to analyze the association.

**Results:**

Overall prevalence of TE, AA, and SD was 61.1, 24.7, and 57.7%, respectively, with female predominance in case of TE and male predominance in case of AA and SD. More than half of the participants had COVID-19-related fear and traumatic stress symptoms. In the multiple logistic regression model, smoking [adjusted odds ratio (aOR) 2.93, 95% CI 1.29–6.65 for AA and aOR 4.19, 95% CI 1.83–9.56 for TE], COVID-19-related fear (aOR 1.70, 95% CI 1.01–2.89 for AA and aOR 2.620, 95% CI 1.25–5.48 for TE), and COVID-19-related traumatic stress symptoms (aOR 1.84, 95% CI 1.08–3.13 for AA, aOR 2.61, 95% CI 1.19–5.68 for TE, and aOR 1.92, 95% CI 1.14–3.25 for SD) were the risk factors of hair fall disorders.

**Conclusion:**

Our study showed that a large number of medical students experienced TE, AA, and SD during the pandemic era. COVID-19-related stress and fear potentially have an association with these diseases.

## Introduction

Psychological stresses have been associated with the development of a variety of skin and hair loss problems globally. Increased psychosocial stress can impact the course of many common “stress-sensitive” skin conditions that lead to actual or perceived exacerbation of the disease (1, 2). Psychological distress, including stress, post-traumatic stress disorder, depression, and anxiety disorders, have all been linked to the emergence or exacerbation of a variety of dermatologic diseases [e.g., psoriasis, atopic eczema, urticaria, alopecia areata (AA), and hair-related problems] (3). The process is mediated by complicated neuroendocrine regulation of inflammatory and related systems. The hypothalamic–pituitary–adrenocortical (HPA) axis plays a crucial role during stressful events and potentially triggers the skin-related disorders (3–5).

Patients with hair diseases, such as telogen effluvium (TE), AA, and scalp seborrheic dermatitis, often reported a low quality of life and increased levels of psychological stress, depression, and anxiety (6–8). The treatment challenges are centered on the role of stress in hair loss and place a premium on the development of appropriate coping mechanisms and the enhancement of patients' quality of life (9). The connection between a stressor and subsequent alterations in the hair growth cycle has resulted in the designation of the brain-hair follicle axis. By promoting the transition of anagen hairs into the telogen process, certain neuropeptides, neurotransmitters, and hormones produced along this brain-hair follicle axis can induce significant alterations in the hair development cycle (7).

Current pandemic, uncertainties, and lockdown circumstances are challenging for the majority of people and contribute to increase psychological suffering worldwide (10, 11). In Bangladesh, university students are considered a vulnerable group for psychological distress as a consequence of coronavirus disease 2019 (COVID-19), and they are experiencing mild to extremely severe psychological stresses during this pandemic (12, 13). In a developing country, such as Bangladesh, where the job market is already scanty after graduation, the uncertainty of academic progress during this pandemic is deteriorating the condition (14). Moreover, medical students have considerably higher rates of psychiatric depression, anxiety, suicidal ideation, and mood disturbances compared with other post-secondary graduate students. Medical students are suffering from severe mental disorders during the COVID-19 pandemic. COVID-19 has caused a variety of negative consequences, including a harmful effect on the mental health of medical students in Bangladesh. A considerable percentage of Bangladeshi medical students is experiencing adverse psychological stresses as a result of the pandemic (15–17).

Different hair-related problems, such as TE, AA, and SD levels, were shown to have increased to some extent during the pandemic (18). A study reported that the patient density for these diseases was substantially greater during the COVID-19 pandemic phase than a year before (19). There is a limited number of literature reviews on this issue. However, TE was reported as a common post-COVID manifestation in infected patients (20). In addition, with these results, it was hypothesized that the psychosocial stress caused by the ongoing pandemic situation may result in exacerbations or the initiation of these hair loss problems. To the best of our knowledge, this is the first study focused on hair diseases associated with COVID-19-related stress in the Southeast Asian region, especially among the Bangladeshi medical students. Hence, this study aimed to report the prevalence and examine the possible association between hair loss problems, especially TE, AA, scalp SD, and COVID-19-related stress among the medical students of Bangladesh. Our research hypothesis was COVID-related stress associated with TE, AA, and SD.

## Materials and Methods

### Study Design and Participants

This was a cross-sectional study that used an online data collection method. All the undergraduate medical students of Bangladesh were the study population. The sample size for the study was calculated by the following formula: $n=\frac{z^{2}p\left(1-p\right)}{d^{2}}$, where z = 1.96 (for 95% confidence level), *p* is the estimated prevalence of hair loss problem, and d is the precision of error. A previous study has reported that the prevalence of hair loss problems during pandemic among Turkish participants was 53.1% (18). Considering this information, the calculated sample size was 404, assuming a 20% non-response rate.

All the undergraduate medical students studying in different government and private medical colleges were our study population. Due to COVID-19 pandemic and lockdown enforced by the government, all the medical colleges were continuing their academic activities online. Hence, we chose the online survey which is already used in a similar study (18). Our inclusion criteria were undergraduate students of both public and private medical colleges of Bangladesh of either sex aged > 18 years and who had access to social media where the survey was circulated. The students who did not use social media (Facebook) and were not attending online classes during the data collection period were excluded from the study. We used a private and common Facebook group for Bangladeshi medical students “Platform” (https://www.facebook.com/groups/platform.organization) where more than 40,000 medical students were joined. We posted the Google Forms link in the group and requested the students to fill it up. The link was active until we reached the targeted number of students.

### Study Instrument

The questionnaire was developed after an extensive literature search in the English language as the medium of learning is English in medical colleges of Bangladesh. The first draft of the questionnaire was reviewed by a consultant psychiatrist and a consultant dermatologist for necessary modification. The questionnaire was pretested among 5% of the study sample (*n* = 20) for content validity and linguistic modification. Internal consistency was assessed by using Cronbach's α. The internal reliability of this study was found to be 0.910, indicating high internal consistency for our scale for this specific sample. The questionnaire has three parts, namely, (i) sociodemographic information, (ii) questions about pre- and post-pandemic TE, AA, and scalp SD, and (iii) information about COVID-19-related stress, and the three separate stress scales were employed to determine the level of stress.

The first dimension of the questionnaire includes demographic features (9 questions), such as name, age, sex, study year, family income, residence, relationship status, body mass index (BMI), and smoking status.

The second dimension includes hair loss problems about pre- and during the pandemic hair loss problems (6 questions), such as TE, AA, and scalp SD. TE is a term that refers to transient hair loss that frequently happens as a result of stress, shock, or a traumatic event. Individuals with this condition lose far more hair than the average 50–100 hairs each day (21).

The AA is a chronic inflammatory complex and widespread autoimmune skin disease that causes sudden non-scarring hair loss on the scalp, face, and sometimes other body parts (22). Acute or chronic psycho-emotional stress or psychological distress may be causing the initiation and progress of AA (23, 24).

The SD is an itchy rash covered in flaky scales condition referred to as dandruff, oily eczema, and seborrheic eczema (25). There is strong evidence of a possible link between stressful life events and episodes of scalp SD (8, 18).

The participants were asked about their scalp hair loss of more than 100 strands a day before and during the pandemic for TE, about focal hair loss-related questions (focal hair loss; also known as ringworm, oval or round para-shaped or wider, sharply circumscribed complete hair loss) for AA, and about the redness of the scalp, dandruff, itching, and oily eczema that caused the complaints before and during the pandemic period for scalp SD.

The third dimension included 3 validated stress scales to determine the level of stress of individuals.

#### COVID-19 Fear Scale

This scale was used to find out the effects of coronavirus on mental health, such as fear, anxiety, and stress (26). This scale is also validated for the Bangladeshi population (27). Using a five-item Likert-type scale, the participants rated their degree of agreement with the claims. Answers included “strongly disagree” to “strongly agree.” The lowest possible score for each question is 1 and the highest possible score is 5. The sum of each item's scores yields a cumulative ranking (ranging from 7 to 35). The higher the ranking, the more concerned and stressed about COVID-19. A score of 20 or higher is considered a fear of COVID-19. The scale demonstrated acceptable internal consistency (Cronbach's α 0.89).

#### Impact of Event Scale Specific for COVID-19

The IES-COVID-19 is a reliable indicator of traumatic stress symptoms due to the COVID-19 epidemic. It is an extension of the commonly used IES (28). This scale has 15 items. Every item is rated on a 4-point scale ranging from “not at all”−0 over “seldom”−1 and “sometimes”−3 to “often”−5. In terms of COVID-19, higher scores suggest a more significant psychological effect of the situation. A score of 26 or higher is considered as developing post-traumatic stress disorder (29). This scale is also previously used among the Bangladeshi population (12). The scale demonstrated acceptable internal consistency (Cronbach's α 0.86).

#### COVID-19 Student Stress Questionnaire

This is a recently created measuring instrument for evaluating the sources of stress associated with the COVID-19 pandemic lockdown among university students (30). There are 7 questions included in the scale related to personal and academic life's stresses during COVID-19 pandemic. It consists of 7 items on a 5-point Likert-type scale ranging from zero (“Not at all stressful”) to four (“Extremely stressful”). The scale provides a Global stress score ranging from 0 to 28. Scores of 6 or below indicate low levels of perceived COVID-19-related Global stress, scores of 7–15 indicate average levels of perceived COVID-19-related Global stress, and scores of 16 or more indicate high levels of perceived COVID-19-related Global stress among university students. The scale demonstrated acceptable internal consistency (Cronbach's α 0.88).

### Ethical Consideration

Ethical approval was obtained from the Ethical Review Committee of the Public Health Foundation, Bangladesh (PHF, BD) (ref. no. 03/2021). Informed written consent was obtained *via* email from participants before inclusion.

### Statistical Analysis

All the statistical analyses were carried out using SPSS version 25.0. For continuous variables, a mean with standard deviation was used, and for nominal variables, a frequency distribution was used. The chi-square test and logistic regression models are used to find out the association between hair diseases and COVID-19-related stress with sociodemographic variables. The variables achieving *p* <0.2 in the bivariate analysis were included in the multiple logistic regression model (Nagelkerke's *R*^2^ = 0.314). Significant results were interpreted as *p* <0.05 with 95% CI.

## Results

Among 404 participants included in our study, female participants were predominant (56%) and most of them (81%) hailed from urban areas. The majority of the participants belonged to the middle (34%) or higher (55%) income families (Table 1).

**Table 1:** Sociodemographic characteristics and COVID-19 pandemic-related stresses of the participants (*n* = 404).

**Characteristics**	***N* (%)**
**Age**	
≤ 22	165 (40.8)
>22	239 (59.2)
**Sex**	
Male	177 (43.8)
Female	227 (56.2)
**Year of study**	
1st year	12 (3.0)
2nd year	50 (12.4)
3rd year	56 (13.9)
4th year	127 (31.4)
5th year	159 (39.4)
**Family income**	
0–20,000	44 (10.9)
20,000–40,000	138 (34.2)
>40,000	222 (55.0)
**Residence**	
Urban	326 (80.7)
Rural	78 (19.3)
**Relationship status**	
Married	24 (5.9)
Unmarried	380 (94.1)
**BMI (kg/m** ^**2**^ **)**	
Underweight (<18.0)	37 (9.2)
Normal weight (18.0–24.9)	255 (63.1)
Overweight (≥25.0)	112 (27.7)
**Smoking status**	
Yes	54 (13.4)
No	350 (86.6)
**COVID-19 related fear**	
Present (≥20)	230 (56.9)
Absent (<20)	174 (43.1)
**COVID-19 related traumatic stress symptoms**	
Present (≥26)	240 (59.4)
Absent (<26)	164 (40.6)
**COVID-19 related stress**	
High (≥16)	143 (35.4)
Average (7–15)	184 (45.5)
Low (≤ 6)	77 (19.1)

It was found that more than half of the participants had COVID-19-related fear and COVID-19-related impact traumatic stress symptoms (56.9 and 59.4% consecutively). In addition, 35.4% of participants had high COVID-19-related student stress.

**Figure 1:**
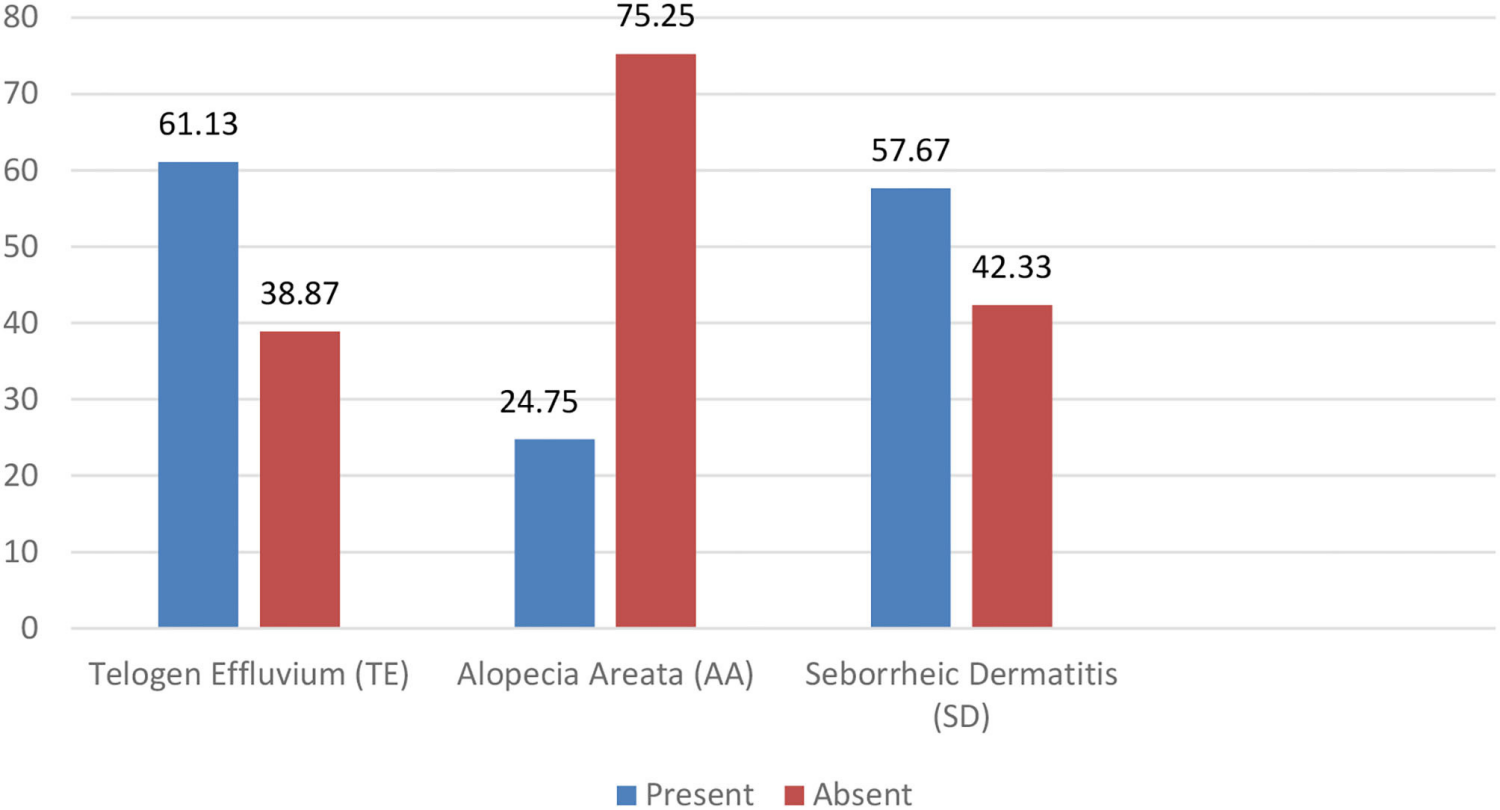
Prevalence of TE, AA, and SD.

In our study, the prevalence of TE, AA, and SD was revealed consequently 61.13% (*n* = 247), 24.75% (*n* = 100), and 57.67% (*n* = 233) (Figure 1).

In our study, female medical students had more prevalence of TE (67.4%); in contrast, SD was more common among male students (59.3%). In addition, TE was more common among first- and third-year students, and AA was more common among fourth-year students. Urban people have more TE than rural people, but, in contrast, rural people have more SD. Persons who smoke have more prevalence in all three conditions (i.e., TE, AA, and SD). Similarly, persons having COVID-19-related fear, COVID-19-related traumatic stress symptoms, and high COVID-19-related student stress have a higher prevalence in all three conditions (i.e., TE, AA, and SD) too. Other prevalence of different groups is shown in Table 2.

**Table 2:** Prevalence of TE, AA, and SD according to participants' characteristics (*n* = 404).

	**Telogen effluvium (TE)**	**Alopecia areata (AA)**	**Seborrheic dermatitis (SD)**
**Age**			
≤ 22	109 (66.1)	32 (19.4)	102 (61.8)
>22	138 (57.7%)	68 (28.5)	131 (54.8)
**Sex**			
Male	94 (53.1)	63 (35.6)	105 (59.3)
Female	153 (67.4)	37 (16.3)	128 (56.4)
**Year of study**			
1st year	5 (41.7)	3 (25.0)	3 (25.0)
2nd year	31 (62.0)	9 (18.0)	33 (66.0)
3rd year	42 (75.0)	16 (28.6)	40 (71.4)
4th year	79 (62.2)	36 (28.3)	71 (55.9)
5th year	90 (56.6)	36 (22.6)	86 (54.1)
**Family income**			
0–20,000	22 (50.0)	7 (15.9)	23 (52.3)
20,000–40,000	81 (58.7)	26 (18.8)	85 (61.6)
>40,000	144 (64.9)	67 (30.2)	125 (56.3)
**Residence**			
Urban	201 (61.7)	77 (23.6)	185 (56.7)
Rural	46 (59.0)	23 (29.5)	48 (61.5)
**Relationship status**			
Married	16 (66.7)	4 (16.7)	15 (62.5)
Unmarried	231 (60.8)	96 (25.3)	218 (57.4)
**BMI**			
Underweight	28 (75.7)	8 (21.6)	20 (54.1)
Normal weight	151 (59.2)	56 (22.0)	142 (55.7)
Overweight	68 (60.7)	36 (32.1)	71 (63.4)
**Smoking status**			
Yes	43 (79.6)	33 (61.1)	40 (74.1)
No	204 (58.3)	67(19.1)	193 (55.1)
**COVID-19 related fear**			
Present	164 (71.3)	80 (34.8)	157 (68.3)
Absent	83 (47.7)	20 (11.5)	76 (43.7)
**COVID-19 related impact traumatic stress symptoms**			
Present	171 (71.3)	82 (34.2)	164 (68.3)
Absent	76 (46.3)	18 (11.0)	69 (42.1)
**COVID-19 related student stress**			
High	110 (76.9)	67 (46.9)	109 (76.2)
Average	98 (53.3)	21 (11.4)	92 (50.0)
Low	39 (50.6)	12 (15.6)	32 (41.6)

In the multiple logistic regression model, it was revealed that male participants had two times more tendency to have AA than female participants (aOR 2.12, 95% CI 1.15–3.93, *p*= 0.016) and smoker participants had four and three times higher risk for developing TE (aOR 4.194, 95% CI 1.839–9.567, *p* = 0.001) and AA (aOR 2.930, 95% CI 1.290–6.658, *p* = 0.010) consequently than nonsmoker participants (Table 3).

**Table 3:** Multiple logistic regression model for the variables associated with TE, AA, and SD (*n* = 404).

	**Telogen effluvium (TE)**		**Alopecia areata (AA)**		**Seborrheic dermatitis (SD)**	
	**aOR (95% CI)**	***p*-value**	**aOR (95% CI)**	***p*-value**	**aOR (95% CI)**	***p*-value**
**Age**						
≤ 22	1.37 (0.72–2.59)	0.329	0.41 (0.18–0.91)	0.029	1.19 (0.64–2.21)	0.573
>22	Ref.		Ref.		Ref.	
**Sex**						
Male	0.41 (0.25–0.68)	0.000	2.12 (1.14–3.92)	0.016	1.05 (0.64–1.71)	0.830
Female	Ref.		Ref.		Ref.	
**Year of study**						
1st year	0.61 (0.14–2.60)	0.513	5.82 (0.97–34.83)	0.053	0.28 (0.06–1.36)	0.117
2nd year	1.01 (0.41–2.49)	0.981	2.03 (0.64–6.42)	0.229	1.57 (0.64–3.82)	0.319
3rd year	1.95 (0.81–4.68)	0.134	4.42 (1.57–12.46)	0.005	2.27 (0.97–5.32)	0.057
4th Year	1.25 (0.70–2.21)	0.445	2.12 (1.04–4.31)	0.037	1.02 (0.58–1.78)	0.933
5th year	Ref.		Ref.		Ref.	
**Family income**						
0–20,000	0.69 (0.33–1.45)	0.337	0.80 (0.30–2.11)	0.653	1.17 (0.56–2.42)	0.670
20,000–40,000	1.01 (0.61–1.66)	0.955	0.79 (0.42–1.48)	0.470	1.75 (1.07–2.87)	0.026
>40,000	Ref.		Ref.		Ref.	
**Residence**						
Urban	1.08 (0.61–1.93)	0.781	0.85 (0.41–1.75)	0.670	0.86 (0.48–1.53)	0.619
Rural	Ref.		Ref.		Ref.	
**Relationship status**						
Married	1.33 (0.50–3.49)	0.563	0.47 (0.13–1.69)	0.251	1.40 (0.53–3.66)	0.487
Unmarried	Ref.		Ref.		Ref.	
**BMI**						
Underweight	2.44 (0.92–6.46)	0.072	0.64 (0.20–2.01)	0.455	0.62 (0.26–1.48)	0.284
Normal Weight	1.085 (0.63–1.84)	0.762	0.735 (0.39–1.35)	0.325	0.76 (0.45–1.28)	0.315
Overweight	Ref.		Ref.		Ref.	
**Smoking status**						
Yes	4.19 (1.83–9.56)	0.001	2.93 (1.29–6.65)	0.010	1.88 (0.86–4.10)	0.112
No	Ref.		Ref.		Ref.	
**COVID-19 related fear**						
Present	1.70 (1.00–2.89)	0.046	2.62 (1.25–5.47)	0.010	1.57 (0.93–2.64)	0.086
Absent	Ref.		Ref.		Ref.	
**COVID−19 related impact traumatic stress symptoms**						
Present	1.84 (1.08–3.13)	0.023	2.61 (1.19–5.68)	0.016	1.92 (1.14–3.25)	0.014
Absent	Ref.		Ref.		Ref.	
**COVID-19 related student stress**						
High	1.35 (0.62–2.90)	0.441	1.28 (0.47–3.46)	0.626	2.10 (0.99–4.47)	0.051
Average	0.64 (0.34–1.21)	0.178	0.33 (0.13–.84)	0.020	0.88 (0.47–1.65)	0.711
Low	Ref.		Ref.		Ref.	

## Discussion

Psychological stress can have an effect on the course of several stress-sensitive skin diseases, such as SD, urticaria, acne vulgaris, TE, AA, and pruritus, causing actual or perceived aggravation of the disease (5, 6, 31). The importance of the brain-skin axis has been repeatedly underlined in cases when there is a complicated link between the brain and the skin (3). Psychological stress influences hypothalamic–pituitary–adrenocortical (HPA) axis hormones as well as stress mediators, such as neuropeptides and cytokine profiles, affecting the immune response which impairs the skin's capacity to adapt to stressful situations. These stress-induced skin alterations might contribute to the worsening of skin disease (32, 33). Furthermore, the link between a stressful situation and any subsequent alterations in the hair development cycle has resulted in the identification of the brain-hair follicle axis. Specific neuropeptides, neurotransmitters, and hormones released along this axis, in particular, can induce substantial alterations in the hair development cycle (1, 7).

The recent outbreak of COVID-19 has exerted tremendous psychological pressure on people of all classes globally (34). These psychological alterations are triggered by fear, stress, anxiety, or uncertainty (35). It has altered the functioning of educational institutions globally, causing significant changes in the everyday lives of students. Due to this unexpected stressful situation, this group has experienced psychological stresses as a result of a dramatic shift in their daily routine (36). Such data suggest that the ongoing psychosocial stress caused by the present pandemic can contribute to the aggravation or onset of prevalent skin and hair diseases at the population level. All of these stressors may enhance the likelihood of developing psychiatric-related dermatologic diseases linked with the COVID-19 pandemic. According to few studies, current pandemic circumstances have already started to alter the course of a variety of skin and scalp diseases, such as TE, AA, and SD in scalp, and other skin diseases (18, 19).

In our study, we selected medical students as a vulnerable group to evaluate pandemic stress associated with hair diseases. Medical students are frequently seen as having a greater level of psychological stress and due to the closure of educational institutions, mental health has been hampered to students all over the country (17). Apart from the general population, medical students are at a higher risk of psychological distress as a result of confronting the new threat of pandemic (16). In addition, in Bangladesh, there is a report of a higher prevalence of depression among medical students, as well as a higher suicidal tendency (15). A large percentage of Bangladeshi medical students are experiencing adverse psychological effects as a result of the pandemic (17). The purpose of this study was to determine the potential association of COVID-19-related stress with hair diseases among medical students in Bangladesh.

In our study, the prevalence of TE, AA, and SD was 61, 25, and 58%, respectively. To the best of our knowledge, there is hardly any previous study on this issue from our country to compare with. However, during this virus outbreak, a number of studies conducted in different countries to explore the pandemic's stress-related hair diseases demonstrate a significant prevalence of hair diseases during this pandemic (18, 37–39).

According to a study conducted in Turkey, the prevalence of TE, AA, and SD was 28, 2.8, and 20%, respectively, in the participants which is comparatively lower than our findings (18). Another Turkish finding revealed that the frequency of AA increased considerably during the COVID-19 pandemic which was hypothesized to be associated with short-term stress (19). Another study from Italy reported that 12.5% of participants experienced relapses of AA during this pandemic (39).

With the global spread of COVID-19 starting in March 2020, physicians and patients have started to report an increase in hair loss (40). Emotional stress associated with COVID-19 pandemic can be associated with TE and SD which usually appear following a stressful incident (18). However, we found that COVID-19-related post-traumatic symptoms and COVID-19-related stress or fear were also associated with all three diseases (i.e., TE, AA, and SD). A previous study has reported that the AA (1.48%) and TE (2.17%) significantly increased during the COVID-19 pandemic along with other skin diseases' dermatological outpatient department. As a result of this investigation, it has been demonstrated that the COVID-19 pandemic sheds fresh light on the emotional stress period associated with hair diseases that may have been induced by psychological imbalances. When compared to the previous year, the percentages of AA and TE increased by 2.83 and 5.51 times, respectively (37). Another study reported that within the first 10 days following the COVID-19 outbreak, SD increased by 4.5% in the dermatology clinic of a tertiary care hospital (37). Some other studies, before the pandemic, identified hair loss and SD as the most common stress-related skin disorders among young adults (41, 42).

In contrast, we have found that smokers had a higher risk for developing TE and AA There is a hypothesis that exogenous nicotine might promote overstimulation of the cellular nicotinic acetylcholine receptors, resulting in receptor desensitization which, in turn, contributes to hair follicle loss by activating keratinocyte-specific programmed cell death pathways (43, 44). Further studies are needed with different subsets of Bangladeshi adult smokers to investigate this particular finding in detail.

Stress has been recognized as an exacerbating element in stress-related skin and hair disorders on several occasions. Such data suggest that the present pandemic's long-term psychological stress may cause exacerbations or the development of skin and hair diseases. However, there is a limitation of data on the interplay of pandemic-related stress and psychological risk factors among people with skin and hair disorders during the COVID-19 pandemic.

As the study's findings indicated a significant degree of stress connected with hair diseases among medical students, we have recommended that the student support system assists and cares for this group. However, this study will benefit a subset of the population that has been exposed to stress for an extended period of time and has developed skin and hair disorders. It is critical to target stress-reduction tactics and treatment facilities in low-resource settings to individuals who are experiencing any level of psychological stress in order to avoid the development of more serious skin disorders associated with stress. Another longitudinal research might be conducted with a cohort of students to determine the degree of stress experienced by students during their undergraduate medical years and the factors that contribute to it.

Nonetheless, these cases suggest that COVID-19 infection, as well as the associated psychological and physiological stress, is capable of causing hair disease. Further research will be required as the pandemic progresses to determine the long-term prevalence and prognosis of hair diseases linked with COVID-19 infection.

This study has several limitations. The major flaw was being a self-reported online survey clinical diagnosis of the hair loss problems was not possible. We can potentially interpret the results largely based on disease symptoms without an exact clinical diagnosis by registered physicians. Moreover, recall bias could not be escaped. Besides, because of its cross-sectional design, causal effect of COVID-19 stress on hair diseases could not be established. Finally, the findings cannot be inferential at the population level as only a specific group of people were included.

## Conclusion

Our study reported that a large number of medical students were experiencing TE, AA, and SD during the pandemic era. COVID-19-related stress and fear potentially have had an effect on these conditions. Further longitudinal and exploratory studies among different subsets of population are needed to enhance our understanding of short- and long-term direct psychological effects on skin and hair disease and to identify effective interventions to address such unique issues. This research opens several doors for psychodermatology to discuss this complex dynamic in the context of low resource settings, such as Bangladesh.

## Data Availability Statement

The raw data supporting the conclusions of this article will be made available by the authors, without undue reservation.

## Ethics Statement

The studies involving human participants were reviewed and approved by the Ethical Review Committee of the Public Health Foundation, Bangladesh (PHF, BD) (ref. no. 03/2021). Informed written consent was obtained via email from participants before inclusion. The patients/participants provided their written informed consent to participate in this study.

## Author Contributions

AM: conceptualization and study design, data collection, and writing—draft preparation. MAR: supervision, reviewing, and editing. TS and SS: data analysis and helping-draft preparation. MGR and MA: data collection and data formatting. MM and MH: reviewing of the draft manuscript for potential intellectual content. All authors read and approved the final manuscript.

## Conflict of Interest

The authors declare that the research was conducted in the absence of any commercial or financial relationships that could be construed as a potential conflict of interest.

## Publisher's Note

All claims expressed in this article are solely those of the authors and do not necessarily represent those of their affiliated organizations, or those of the publisher, the editors and the reviewers. Any product that may be evaluated in this article, or claim that may be made by its manufacturer, is not guaranteed or endorsed by the publisher.
